# Development of a Peptide-Based Nano-Sized Cathepsin B Inhibitor for Anticancer Therapy

**DOI:** 10.3390/pharmaceutics15041131

**Published:** 2023-04-03

**Authors:** So-Hyeon Park, Jun-Hyuck Lee, Seong-Bin Yang, Dong-Nyeong Lee, Tae-Bong Kang, Jooho Park

**Affiliations:** 1 Department of Applied Life Science, BK21 Program, Graduate School, Konkuk University, Chungju 27478, Republic of Korea; 2 Center for Metabolic Diseases, Konkuk University, Chungju 27478, Republic of Korea

**Keywords:** cathepsin B, bioconjugate, anticancer therapy, nanoparticle, peptide drug

## Abstract

Numerous cathepsin B inhibitors have been developed and are under investigation as potential cancer treatments. They have been evaluated for their ability to inhibit cathepsin B activity and reduce tumor growth. However, they have shown critical limitations, including low anticancer efficacy and high toxicity, due to their low selectivity and delivery problems. In this study, we developed a novel peptide and drug conjugate (PDC)-based cathepsin B inhibitor using cathepsin-B-specific peptide (RR) and bile acid (BA). Interestingly, this RR and BA conjugate (RR–BA) was able to self-assemble in an aqueous solution, and as a result, it formed stable nanoparticles. The nano-sized RR–BA conjugate showed significant cathepsin B inhibitory effects and anticancer effects against mouse colorectal cancer (CT26) cells. Its therapeutic effect and low toxicity were also confirmed in CT26 tumor-bearing mice after intravenous injection. Therefore, based on these results, the RR–BA conjugate could be developed as an effective anticancer drug candidate for inhibiting cathepsin B in anticancer therapy.

## 1. Introduction

Cathepsin B is an important target in cancer research and has been widely studied for the past 20 years. It is a cysteine peptidase of the lysosomal cysteine protease family, which plays an important role in proteolysis. In the cathepsin family, cathepsin enzymes, such as cathepsin A, B, C, D, E, F, G, H, K, L, O, S, W, and Z, contribute to the development of several diseases, participating in multiple biological processes, such as apoptosis, autophagy, inflammation, and extracellular matrix remodeling [1]. Cathepsin B is involved in many physiological processes, such as cancer migration, angiogenesis, and metastasis [2,3]. It is highly upregulated in certain malignant lesions and several cancers, including colorectal, esophageal, and liver carcinoma [4,5]. The overexpression of cathepsin B in cancer patients is correlated with invasiveness or tumor metastasis, and it has been studied as a tumor biomarker for a variety of cancers [6]. The role of cathepsin B in tumorigenicity or tumor metastasis has been extensively studied in recent mechanistic research [7]. Targeting and controlling cathepsin B in cancer patients have opened a new avenue for designing pharmacological approaches in cancer treatment [8].

Investigations into the cathepsin B enzyme in tumor tissue have attracted a lot of attention, since it plays a pivotal role in tumor invasion and metastasis. Much recent pharmacological research has been focused on targeting overexpressed cathepsin B in cancer cells [9,10]. Researchers have mainly utilized the peptide-cleavage ability of cathepsin B enzymes [11,12,13,14]. Cathepsin B enzymes recognize and cleave specific peptide sequences, such as Arg-Arg, Val-Ala, Phe-Lys, Gly-Leu-Phe-Gly, Gly-Phe-Leu-Gly, and Ala-Leu-Ala-Leu [12,15,16]. Based on this, various drug conjugates and prodrugs have been developed in pharmacological research and actively researched as new drug candidates in recent years [17,18,19]. In the drug development of new antibody–drug conjugates (ADCs) and peptide–drug conjugates (PDCs), related pharmacological research is actively progressing, and some of these conjugates have reached clinical trials [10,20,21]. Because of the overexpression and cellular activity of cathepsin B observed in many cancers, these tumor-specific prodrug studies will continue in cancer research [22,23,24,25].

Cathepsin B inhibitors are being actively investigated as potential anticancer agents due to their role in cancer cell invasion and migration. Targeting and inhibiting cathepsin B in cancer cells could have significant therapeutic implications [26,27]. Several small inhibitors of cathepsin B have been developed and are currently in various stages of preclinical and clinical evaluations [28,29]. Some examples of cathepsin B inhibitors that have been studied include CA074, E6446, and CA045. The results of early studies have shown promising efficacy in reducing tumor growth and spread, but further clinical trials are necessary to fully assess their safety and effectiveness for cancer treatment. However, their anticancer effects do not appear to be strong enough to completely inhibit or eliminate cancers in animal models due to their diverse functions. Many potential cathepsin B inhibitors have been developed, but none of them have shown clinically available strong anticancer effects, and there currently are no FDA-approved cathepsin B inhibitors for cancer treatment [7,30].

**Figure 1:**
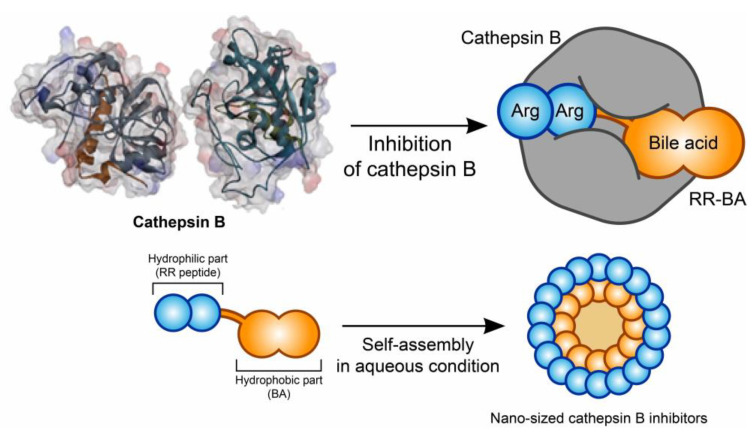
Schematic image of self-assembled amphiphilic bile-acid-based cathepsin B inhibitory nanoparticles. RR–BA molecules can be self-assembled in aqueous conditions from RR peptides and bile acid due to their amphiphilicity. RR–BA molecules could act as cathepsin B inhibitors because they have a cathepsin B binding site.

Here, we developed a cathepsin B inhibitor using a peptide and bile acid for anticancer therapy. Among the peptide sequences that cathepsin B can recognize, positively charged Arg-Arg (RR) was used as a cathepsin-B-specific peptide without any linkers. A hydrophobic bile acid (BA) was coupled via amide linkages to further enhance the inhibitory effects of the peptides. Out of several types of bile acids, ursodeoxycholic acid (UDCA), a safe drug approved by the US Food and Drug Administration (FDA), was used for this study. The nano-sized Arg-Arg and BA conjugate (RR–BA) can be self-assembled in an aqueous solution, forming nanoparticles due to the strong positive charge and the hydrophobicity of bile acids (Figure 1). This pharmaceutical substance has shown promising results in mouse colorectal cancer (CT26) cell treatment and animal experiments. This type of pharmaceutical therapy could be a potential option for cancer treatment.

## 2. Materials and Methods

### 2.1. Materials

The C-terminal modified RR peptide (Arg-Arg ethylamide, RR-NHEt; 357.4 Da) was synthesized by ANYGEN (Gwangju, Republic of Korea). Acetonitrile (ACN), antibiotic antimycotic solution, a cathepsin B activity assay kit, *N*,*N*-diisopropylethylamine (DIPEA), anhydrous *N*,*N*-dimethylformamide (DMF), Dulbecco’s Modified Eagle’s Medium (DMEM), diethyl ether, *N*-(3-dimethylaminopropyl)-*N*′-ethylcarbodiimide hydrochloride (EDC), *N*-hydroxysuccinimide (NHS), methanol, phosphate-buffered saline (PBS), trifluoroacetic acid (TFA), and ursodeoxycholic acid (UDCA) were purchased from Sigma-Aldrich (Milwaukee, WI, USA). Fetal bovine serum (FBS) was obtained from Gibco (Waltham, MA, USA). An Ez Cytox kit was obtained from DoGenBio (Seoul, Republic of Korea). Neutral buffered formalin (10%) was purchased from HuBenTech (Damyang, Republic of Korea). A Pierce BCA protein assay kit was obtained from Thermo Fisher Scientific (Waltham, MA, USA). Chloroform was purchased from DAEJUNG (Siheung, Republic of Korea).

### 2.2. Synthesis

The cathepsin-B-specific and hydrophilic RR peptide (RR-NHEt) was synthesized by ANYGEN. Then it was directly conjugated with ursodeoxycholic acid (UDCA) via a one-step amide bond coupling reaction. A total of 50 mg of RR peptide (RR-NHEt) was dissolved in anhydrous DMF, and then 27.5 mg of ursodeoxycholic acid was added to the peptide-containing DMF. After 5 min stirring, 8.1 mg of N-Hydroxysuccinimide (NHS) and 40.2 mg of N-(3-Dimethylaminopropyl)-N′-ethylcarbodiimide hydrochloride (EDC) were added to the solution for the coupling reaction. Then, 4.9 µL of *N*,*N*-diisopropylethylamine (DIPEA) was further mixed in to avoid salt’s effects. The solution was continuously stirred at 600 rpm at room temperature for 24 h. After one day of incubation, the product was slowly precipitated in 14 mL of cold ether/chloroform (6:1) co-solvent twice. The precipitant was collected using a centrifuge at 2000 rpm for 4 min. After removing the upper solvent, the remaining solvent in the white powder was further removed for 25 min using a vacuum evaporator, then 5 mL of distilled water was added, and the sample was freeze-dried for 48 h. Next, the powder was dissolved in distilled water (1 mL) and then further purified via Sep-Pak C18 Plus cartridges (Waters, Milford, MA, USA) using distilled water (100%) and acetonitrile/water (50/50%) cosolvent. The solution was finally freeze-dried to obtain a white powder.

### 2.3. Characterizations

#### 2.3.1. RP-HPLC Analysis

The purity of the final product was confirmed via reverse-phase high-performance liquid chromatography (RP-HPLC) (1200 series, Agilent Technologies, Santa Clara, CA, USA). All analyses were carried out on an Eclipse Plus C18 reverse column (3.5 μm 4.6 × 150 mm; Agilent) by gradient elution with distilled water (90% to 10%) and acetonitrile (10% to 90%) containing 0.1% trifluoroacetic acid (TFA) as mobile phases at a flow rate of 1.0 mL/min. The product was measured using an ultraviolet–visible (UV–Vis) detector at 224 nm. Additionally, in all processes of synthesis, the mixture was observed via normal-phase thin-layer chromatography (TLC) with a mobile phase CH_3_OH/CH_3_COCH_3_/CH_3_COOH (5:3:2, *v*/*v*/*v*).

#### 2.3.2. HPLC-MS and NMR Measurement

For mass spectroscopy, liquid chromatography–mass spectrometry (LC–MS; Agilent Technologies 1260 infinity series, Santa Clara, CA, USA) was used to identify the final product (RR–BA). In the scan mode, atmospheric pressure ionization-electrospray (API-ES) techniques were used to detect the molecular weights of RR–BA molecules with a capillary voltage of 3000 V with drying gas (350 °C). The final products were also confirmed via one-dimensional proton nuclear magnetic resonance (1D proton NMR) analysis, using a 500 MHz NMR spectrometer (JEOL, JNM-ECZ500R/S1, Tokyo, Japan) at a concentration of 5 mg/600 μL with dimethylsulfoxide (DMSO-d_6_).

### 2.4. Nanoparticle Analysis

The nanoparticle formation and size distribution of RR–BA were evaluated via dynamic light scattering (DLS; Zeta sizer Nano, Malvern Instruments, Worcestershire, UK) analysis. The RR–BA materials were dissolved in filtered normal saline (0.9% NaCl) at 1 mg/mL for DLS measurement. The zeta potential value of RR–BA (1 mg/mL) in saline was also evaluated using the Zetasizer Nano method. The spherical morphology of the self-assembled RR–BA nanoparticles (1 mg/mL) in distilled water was observed via transmission electron microscopy (TEM; EVO MA 10, Carl Zeiss, Oberkochen, Germany).

### 2.5. Cathepsin B Inhibition Assay

Mouse colorectal cancer (CT26) cells were purchased from the Korean Cell Line Bank (Seoul, Republic of Korea). They were cultured in 10% FBS containing high-glucose DMEM medium and then collected by scraping, with treatment of cell lysis buffer, using the cathepsin B activity assay kit (Sigma-Aldrich, Milwaukee, WI, USA). The cell solution was centrifuged at 13,000 rpm for 15 min at 4 °C, followed by 5 min incubation on ice for the collected supernatants. After determining the protein concentration for the collected supernatant using a Pierce BCA protein assay kit (Thermo Fisher Scientific), 50 µg of cell lysate was combined with cathepsin B reaction buffer (cathepsin B activity assay kit) in a 96-well black plate. Cathepsin B inhibitor (cathepsin B activity assay kit, 1 mM) was used as a control to inhibit cathepsin B activity in CT26 cells and for comparison with different treatments of RR–BA (1, 5, or 10 mM). In addition, RR peptide (1, 5 or 10 mM) was also used to compare the inhibition effect of cathepsin B activity with RR–BA. After the addition of a fluorogenic cathepsin B substrate, Ac-RR-AFC (cathepsin-B-specific substrate in the cathepsin B activity assay kit, 1000 µM), the plate was incubated for 2 h at 37 °C. Then, the fluorescence of the wells was measured using a microplate reader (SpectraMax M2) at wavelengths of λ_Ex_ = 400 nm/λ_Em_ = 505 nm.

### 2.6. In Silico Computer Simulation for Molecular Binding

The molecular structure of RR–BA was drawn using ChemDraw Professional 20.1.1.125 (PerkinElmer Inc, Waltham, MA, USA). Molecular docking was performed with AMDock (Assisted Molecular Docking) software, version 1.5.2, using cathepsin B protein (PDB: 1QDQ) [31] and a CHARMm (Chemistry at Harvard Macromolecular Mechanics) force field. The active site of cathepsin B for inhibitors was used as the binding site based on the existing inhibitors in PDB (1QDQ). The best pose was selected from the 10 recorded poses and visualized using Discovery Studio 2022 (BIOVIA, San Diego, CA, USA) software. Then, binding energy analysis was conducted using the calculated binding energy protocol with in-suit ligand minimization. For molecular dynamics (MD) simulation, a GBSW (Generalized Born with simple switching) implicit solvent model was used to measure the binding stability of RR–BA. MD simulation was performed using the standard dynamic cascade protocol and observed as an NVE ensemble under the GBSW. During simulation, the intermolecular interaction energy between RR–BA and cathepsin B was calculated from the analyzed trajectory for the 100 ps production process.

### 2.7. In Vitro Cytotoxicity Study

The cytotoxicity of the RR–BA conjugates was evaluated using a mouse-derived colon carcinoma cell line (CT26 cells). The CT26 cells were cultured with high-glucose Dulbecco’s Modified Eagle’s Medium (DMEM) containing 10% fetal bovine serum and 1% antibiotic/antimycotic solution. Cells were seeded in 96-well plates at a concentration of 1.0 × 10^4^ cells/well (for 24 h) or 5.0 × 10^3^ cells/well (for 48 h). First, they were preincubated at 37 °C in a 5% CO_2_ incubator for 1 h. Then, the cells were further incubated with 0.1–1000 µM of RR, BA, or RR–BA for 24 and 48 h. The treated wells (n = 6) were evaluated using an Ez Cytox cell viability assay kit (Daeil Lab Service, Seoul, Republic of Korea) and a microplate reader (SPECTROstar^Nano^, BMG LABTECH, Ortenberg, Germany) at wavelengths of 450/600 nm. The cell viability was calculated from the measured UV absorbance values of samples compared with that of the control blank.

### 2.8. In Vivo Antitumor Effect of RR–BA Nanoparticles in Tumor-Bearing Mice

All animal experiments were performed according to the standard regulations of the Konkuk University Institutional Animal Care and Use Committee (Ref: no. KU22078). A total of 5.0 × 10^6^ cells/50 µL of CT26 cells were inoculated in the back of 6-week-old male BALB/c mice (Orient Bio, Seungnam, Republic of Korea). After approximately 1 week, when the tumor volume had grown to approximately 50–80 mm^3^ (largest diameter × smallest diameter^2^ × 0.52), the tumor-bearing mice were divided into four groups. Then, the mice were intravenously (i.v.) injected or administered per os (p.o.) with normal saline, RR–BA (15 mg/kg/day, i.v.), RR peptide (7.32 mg/kg/day, i.v.), or BA (8.04 mg/kg/day, p.o.) for 20 days. BA (ursodeoxycholic acid) was administered orally due to solubility problems. The body weight and tumor volume of the treated mice were measured every other day using a digital caliper (ASIMETO, Mooresville, NC, USA). After 20 days of treatment, the tumors were extracted from the sacrificed mice and then fixed with 10% neutral buffered formalin (HuBen Tech, Republic of Korea). The isolated tumor tissues were stained with hematoxylin and eosin solution (H&E), and then sliced for histological observation using an inverted microscope (ECLIPSE Ts2, Nikon, Japan). In addition, the isolated tumor tissues were evaluated via terminal deoxynucleotidyl transferase dUTP nick end labeling (TUNEL) staining. The result was quantified using Stardust (Fiji image processing) and ImageJ (USA National Institutes of Health) [32].

## 3. Results and Discussion

### 3.1. Synthesis and Characterization of RR–BA as a Cathepsin B Inhibitor

**Figure 2:**
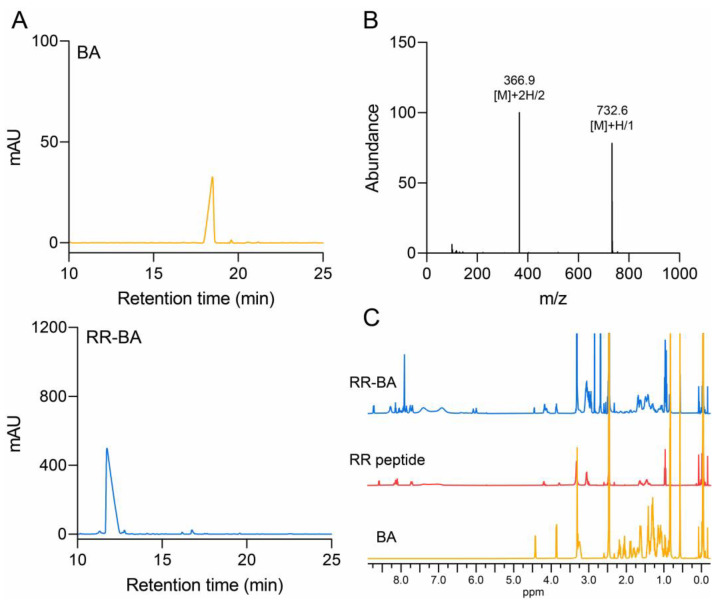
Characterization of RR–BA as a cathepsin B inhibitor. (**A**) The RP-HPLC result showed not only the synthesis of the RR–BA molecule but also that it was endowed with hydrophilicity. (**B**) HPLC-MS chromatogram of RR–BA (MW = 731.5) in positive ionization mode. (**C**) The 1D proton NMR spectrum of RR–BA, RR peptide, and BA in DMSO-d6 was measured using a 500 MHz NMR spectrometer.

Because of the tendency of cathepsin B to be overexpressed in many cancer cells, its inhibitors have potential value as anticancer agents [11,27]. We expected that the newly designed self-assembled cathepsin B inhibitor from this would be able to overcome the current limitations of cytotoxic agents for cancer therapy. In order to prepare a peptide-based biomolecule that could bind to cathepsin B, the cathepsin-B-specific hydrophilic peptide sequence of Arg-Arg (RR) was directly conjugated to the hydrophobic ursodeoxycholic acid (BA). This was able to form a peptide–drug conjugate (PDC) because ursodeoxycholic acid is a safe US-FDA-approved drug. The carboxylic end of the RR peptide was blocked by ethylamide (NHEt) to prevent unnecessary side reactions. The amphiphilic Arg-Arg peptide (RR) and bile acid (BA) conjugate (RR–BA) was synthesized via a one-step synthetic protocol using an EDC/NHS reaction. They were reacted in DMF with *N*,*N*-diisopropylethylamine (DIPEA) at 600 rpm at room temperature for 24 h (Figure S1). The primary amine group of RR-NHEt was directly bound to the carboxylic acid group of the UDCA. After the reaction, the synthesized RR–BA was purified using a cold ether/chloroform (6:1) co-solvent (twice) in a centrifuge at 2000 rpm for 4 min and Sep-Pak C18. Finally, the purified RR–BA was confirmed using reverse-phase HPLC (RP-HPLC) (Figure 2A). The synthesized RR–BA was further confirmed using liquid chromatography–mass spectrometry (LC–MS, m/z calculated: 731.5, observed: 732.6 [M + H^+^] and 366.9 [(M + 2H^+^)/2]) (Figure 2B). In addition, the chemical structure of RR–BA was analyzed via ^1^H NMR based on the observed BA and RR peptide peaks of 0.8–2.5 ppm and 6.5–8.5 ppm (Figure 2C).

### 3.2. Nano Characterization of RR–BA

**Figure 3:**
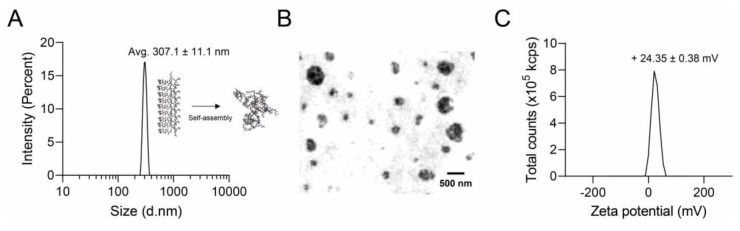
Nano-characterization of RR–BA molecules. (**A**) RR–BA nanoparticles in aqueous conditions with a Z-average size of 283.6 nm, polydispersity index (PDI) of 0.1424, and size (intensity) of 307.1 ± 11.1 nm. (**B**) Transmission electron microscopy (TEM) image of RR–BA nanoparticles. (**C**) Zeta potential distribution of RR–BA, showing a positively charged surface.

The nanoparticle structure of RR–BA in saline was characterized using dynamic light scattering (DLS). As expected from the amphiphilic property of RR–BA molecules, they formed self-assembled nanoparticles with a Z-average size of 283.6 nm (intensity) (Figure 3A) or 286.5 ± 15.9 nm (number) (Figure S2) in saline, without any nano-sized carriers. This is because the RR–BA molecules have suitable hydrophilicity from the peptides and hydrophobicity from the BA moiety. The distinct nano-sized morphology of self-assembled RR–BA nanoparticles in water was clearly observed via transmission electron microscopy (TEM) (Figure 3B). The zeta potential of the RR–BA nanoparticles in saline was also measured as +24.35 ± 0.38 mV due to the presence of highly positively charged RR peptides on the nanoparticle surface (Figure 3C). The zeta potential of various concentrations of RR–BA (0.25, 0.5, 1, 2, or 4 mg/mL) dissolved in normal saline (0.9% NaCl) was also measured (Figure S3).

### 3.3. Computer Simulation of RR–BA with Cathepsin B

**Figure 4:**
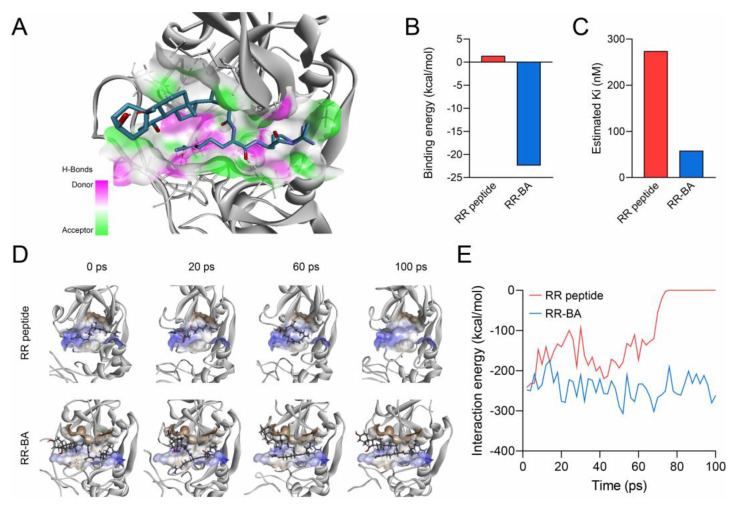
(**A**) Interactions of the docked RR–BA molecule with amino acid residues of cathepsin B. (**B**) Calculated binding energy of RR–BA and RR peptide in docked conformation. (**C**) Estimated potential Ki value from docking conformation using AMDock Software, version 1.5.2. (**D**) Dynamic conformations of the RR–BA and cathepsin B complex during 100 ps MD simulation. (**E**) Calculated molecular interaction energy of the RR–BA and cathepsin B complex for 100 ps MD simulation.

To confirm the molecular binding of RR–BA with the cathepsin B protein, molecular docking was conducted to evaluate the potential of RR–BA as an inhibitor of cathepsin B. RR–BA was stably and well bound to the potential binding site of cathepsin B (Figure 4A) compared with the RR peptide itself (Figure S4). The docking score for the bound RR–BA to cathepsin B was found to be –8.3 kcal/mol, while that of RR alone was –6.3 kcal/mol. A further analysis using the Generalized Born with simple switching (GBSW) model revealed that RR–BA molecules possessed a binding energy of –22.44 kcal/mol, which was more than about 20 times lower than that of RR (1.36 kcal/mol) (Figure 4B). The molecular interactions were found to involve various interactions between the amine group of Arg in RR–BA and Glu245 in cathepsin B with other amino acids (Figure S5). The bound BA molecule exhibited multiple interactions between its ring region with Met196, His111, and Val176 in the cathepsin B enzyme via alkyl and pi-alkyl interactions, which enhanced the binding affinity. Finally, the predicted potential inhibition concentrations (Ki) were 58 nM for RR–BA and 273 nM for the RR peptide, showing that RR–BA could be a more effective inhibitor (Figure 4C).

A 100 ps molecular dynamics (MD) simulation with the bound molecule complex was performed to investigate the binding stability of RR–BA in the catalytic pocket of cathepsin B. The RR–BA and cathepsin B molecules were simulated after adding the effect of the implicit water molecule with cathepsin B. During a 100 ps MD simulation, the dissociation of the RR molecule was observed at 72 ps, while the RR–BA molecule maintained its position by forming a stable complex at the binding site of cathepsin B (Figure 4D). This indicated the increased overall stability of RR–BA in the catalytic pocket of cathepsin B. To confirm the additional binding affinity in the MD simulation, the interaction energy with the protein and RR and RR–BA was investigated for 100 ps. The interaction energy of RR and cathepsin B was averaged to be –114.55 kcal/mol for 100 ps, while the interaction energy of RR–BA and cathepsin B was found to be –241.68 kcal/mol (Figure 4E). Therefore, it was observed through computer simulations that the RR–BA molecule could more stably bind to cathepsin B than RR peptides.

### 3.4. Inhibition Assay Using a Cathepsin B Activity Assay Kit

**Figure 5:**
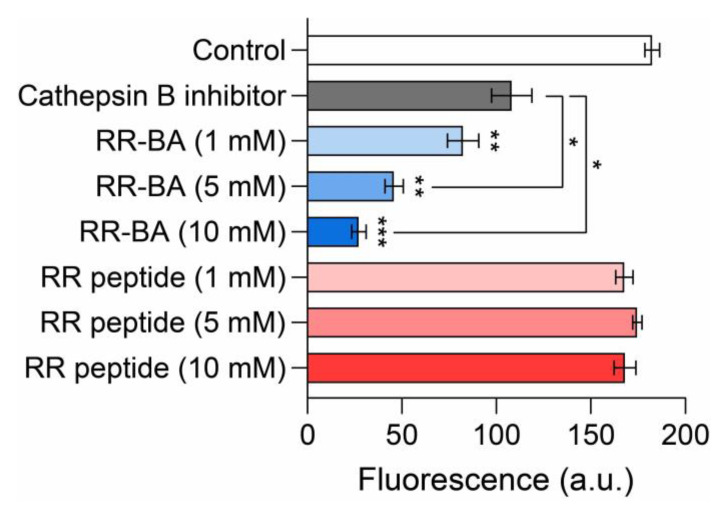
Cathepsin B expression level analysis. Cathepsin B activity was measured in CT26 cells that were treated with the control (Ac-RR-AFC), cathepsin B inhibitor (from the cathepsin B activity assay kit) as a positive control, or various concentrations of RR peptide and RR–BA for 2 h. The cathepsin B enzymatic activity is presented in fluorescence units (a.u.). * *p* < 0.05, ** *p* < 0.01, *** *p* < 0.001. Results represent means ± S.D.

A cathepsin B activity assay was conducted to confirm RR–BA as a cathepsin B inhibitor. It was performed using CT26 tumor cells as cathepsin B donors, with Ac-RR-AFC (the cathepsin B substrate, 1 mM) as a fluorogenic substrate as the control. A total of 1 mM of the cathepsin B inhibitor from a cathepsin B activity assay kit (Sigma-Aldrich; Milwaukee, WI, USA) was used as a positive control. Various concentrations (1, 5 or 10 mM) of RR peptide or RR–BA were used to compare cathepsin B inhibition with the cathepsin B inhibitor. As expected, RR–BA significantly inhibited the enzyme activity of cathepsin B as a cathepsin B inhibitor by binding to cathepsin B instead of Ac-RR-AFC (Figure 5). The fluorescence from Ac-RR-AFC was reduced in a concentration-dependent manner with RR–BA. Interestingly, it was confirmed that the fluorescence from Ac-RR-AFC was reduced more than with the cathepsin B inhibitor in the cathepsin B activity assay kit.

### 3.5. Cathepsin-B-Dependent Cytotoxic Effect

**Figure 6:**
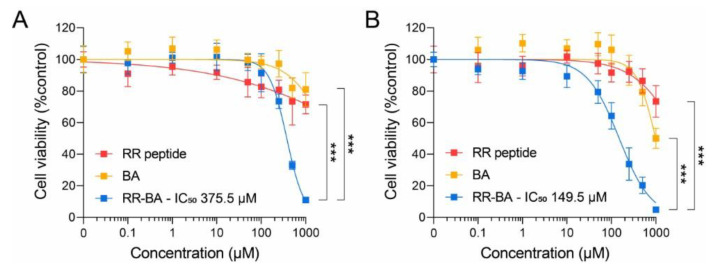
Cell viability and corresponding IC50 values of CT26 tumor cells in the concentration range of 0.1–1000 µM RR–BA, RR peptides, or BA were measured after 24 h (**A**) and 48 h (**B**) incubation. The 24 h cytotoxicity IC50 value of RR peptide and BA is NA and RR–BA is 375.5 µM (**A**). The 48 h cytotoxicity IC50 value of RR peptide and BA is NA and RR–BA is 149.5 µM (**B**). *** Denotes statistically significant differences in a comparison of RR–BA with RR peptides and BA (*** *p* < 0.001). Results represent means ± S.D.

sTo evaluate the cathepsin-B-based cancer cell cytotoxicity of RR–BA nanoparticles, we carried out an in vitro cytotoxicity assay in CT26 colon carcinoma cells for 24 or 48 h. When the CT26 cells were given different concentrations of RR–BA (0.1–1000 µM) for 24 h, the RR–BA showed tumor cell cytotoxicity compared with the RR peptides and BA (Figure 6A). Furthermore, the dose-dependent cytotoxicity of RR–BA (0.1–1000 µM) revealed more cytotoxicity against the CT26 tumor cells after 48 h incubation than the RR peptides and BA from 10 µM (Figure 6B). The cytotoxic effect of RR–BA was not strong, because the targeting of cathepsin B alone in cells was not enough to kill tumor cells due to its diverse functions [27]. Nevertheless, the inhibition of cathepsin B in cancer cells acts apoptotically by affecting Bak, cytochrome C, caspase 9, and X-linked inhibitor of apoptosis protein (XIAP) [33].

### 3.6. In Vivo Anticancer Effect of RR–BA in Tumor-Bearing Mice

**Figure 7:**
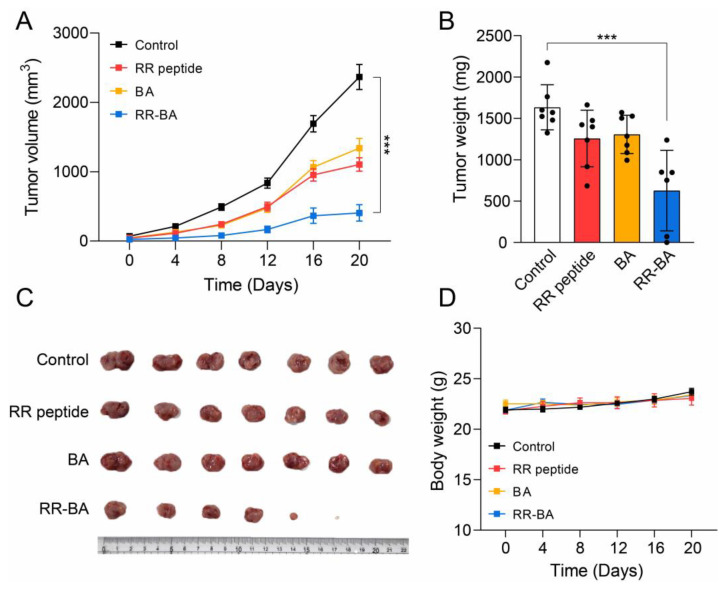
Tumor inhibitory effect in the CT26 tumor-bearing mice. (**A**) Tumor volume change. (**B**) Tumor weights on the last day of the experiment. (**C**) Isolated tumors after sacrifice. (**D**) The body weights of tumor-bearing mice. *** Denotes statistically significant differences in comparison with the saline-treated group (control).

The self-assembling nature of RR–BA nanoparticles might improve their tumor targeting effect on tumor tissues via an enhanced permeability and retention (EPR) effect [34,35,36]. To evaluate the anticancer effect of RR–BA nanoparticles in an animal model, murine CT26 cells and BALB/c mice were used. First, the CT26 tumor-bearing mouse model was prepared by the inoculation of CT26 cells on the flanks of mice. Then, RR–BA (15 mg/kg/day) and RR peptides (7.3 mg/kg/day) were intravenously injected, and BA (8.0 mg/kg/day) was administered orally, to confirm their therapeutic efficacy. BA (ursodeoxycholic acid), a widely used FDA-approved oral drug, was administered orally because it is not soluble in saline. All the administrations were performed daily for 20 days. As shown in Figure 7A, the tumors treated with RR–BA (406 ± 237 mm^3^, n = 6) exhibited significant tumor inhibition compared with those treated with BA (1340 ± 239 mm^3^, n = 7), RR peptides (1104 ± 200 mm^3^, n = 7), or the control group (2366 ± 350 mm^3^, n = 7). After the tumor-bearing mice were sacrificed, the isolated tumor weight of the mice treated with RR–BA (537 ± 423 mg) was significantly lower than that of the BA (1306 ± 195 mg), RR peptide (1257 ± 266 mg), and untreated control (1634 ± 191 mg) groups (Figure 7B). The relative size of the resected tumor tissues was then measured to compare the therapeutic effects (Figure 7C). In addition, there was no noticeable change in the body weight of the RR–BA, BA, RR peptide, or untreated control tumor-bearing mice over the 20 days (Figure 7D), meaning no significant systemic toxicity. These results indicate that RR–BA showed a remarkable anticancer effect in mice without severe toxicity. This strong anticancer effect of RR–BA seems to be due not only to the inhibitory effect on cathepsin B, but also to other complex factors related to BA, because the other treated group also experienced anticancer effects [37,38,39].

**Figure 8:**
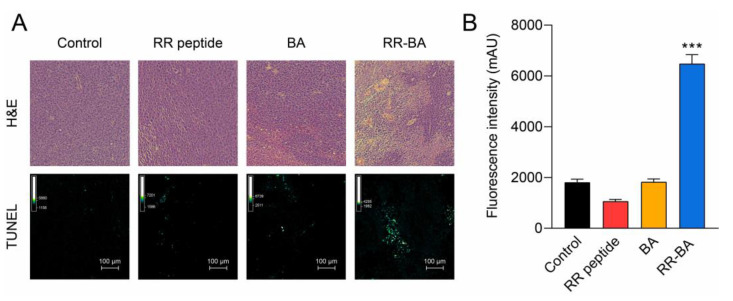
Tumor tissue analysis. (**A**) Images of H&E-stained tumor tissue were histologically analyzed. (**B**) The fluorescence intensity of TUNEL was analyzed quantitatively (*** *p* < 0.001).

Furthermore, an H&E staining analysis of the isolated tumors was conducted to confirm apoptosis and cell death in the tumor tissue. The results showed extensive destruction in the tumor tissue in the RR–BA-administered mice (Figure 8A). Additionally, a TUNEL (terminal deoxynucleotidyl transferase dUTP nick end labeling) assay of the sacrificed tumor tissue showed the detection of DNA breaks generated during the apoptosis of tumor tissue in the RR–BA treated mice (Figure 8A). The anticancer effects of RR–BA were further quantified using Stardust Fiji processing and ImageJ. As a result, more TUNEL-positive signals were observed in RR–BA treated tumor tissue, as shown in Figure 8B.

## 4. Conclusions

The novel nano-sized RR–BA conjugates developed in this paper showed remarkable therapeutic potential and low toxicity. Cathepsin B is a lysosomal protease that is involved in tumor progression and is a promising therapeutic target for cancer. Inhibiting cathepsin B has the potential to slow down tumor growth. The development of this type of nano-sized cathepsin B inhibitor may be a promising approach for future anticancer therapy. In this study, we designed a new drug candidate based on the concept of peptide-drug conjugates and nanoformulation. The synthesized RR–BA molecules showed significant cathepsin B inhibitory ability and anticancer effects through cathepsin-B-specific peptides and hydrophobic BA. However, like other cathepsin B inhibitors, it has a limitation in that its anticancer effect is not strong enough to completely suppress the tumor, but its therapeutic efficacy as an anticancer drug candidate is sufficient. In addition, because of its great value as an oral agent, there are areas to be studied in the future. Therefore, RR–BA could be a new potential anticancer drug candidate for anticancer therapy. Based on these results, we conclude that RR–BA is a novel anticancer agent with cathepsin B inhibitory efficiency and low toxicity for biomedical applications.

## Data Availability

Not applicable.

## Supplementary Materials

The following supporting information can be downloaded at: https://www.mdpi.com/article/10.3390/pharmaceutics15041131/s1, Figure S1: The chemical synthesis of RR–BA conjugate by using RR peptide and bile acid; Figure S2: Size distribution analysis by dynamic light scattering (DLS); Figure S3: Determining the zeta potential of RR–BA; Figure S4: In silico docking position of RR–BA at cathepsin B binding site; Figure S5: Molecular interaction analysis of RR peptide (left) and RR–BA (right) with cathepsin B.
