# Supplementary material for: Development of a Peptide-Based Nano-Sized Cathepsin B Inhibitor for Anticancer Therapy
Source: Pharmaceutics. 2023 Apr 3;15(4):1131. doi: 10.3390/pharmaceutics15041131 (PMC10141979; doi:10.3390/pharmaceutics15041131)
Supplement: Supplementary file 1 [file pharmaceutics-15-01131-s001.zip › pharmaceutics-2268258-supplementary.pdf]

SUPPORTING INFORMATION

Development of a Peptide-based Nano-sized Cathepsin B Inhibitor for Anticancer Therapy

*So-Hyeon Park[1‡], Jun-Hyuck Lee[1‡], Seong-Bin Yang[1], Dong-Nyeong Lee[1], Tae-Bong Kang[1] and Jooho Park[1, 2]**

[1]Department of Applied Life Science, Graduate School, BK21 Program, Konkuk University, Chungju 27478, Republic of Korea

[2]Center for Metabolic Diseases, Konkuk University, Chungju 27478, Republic of Korea

[‡]These authors contributed equally to this work

***Corresponding author:** (Phone) +82-43-840-3574; (E-mail) pkjhdn@kku.ac.kr

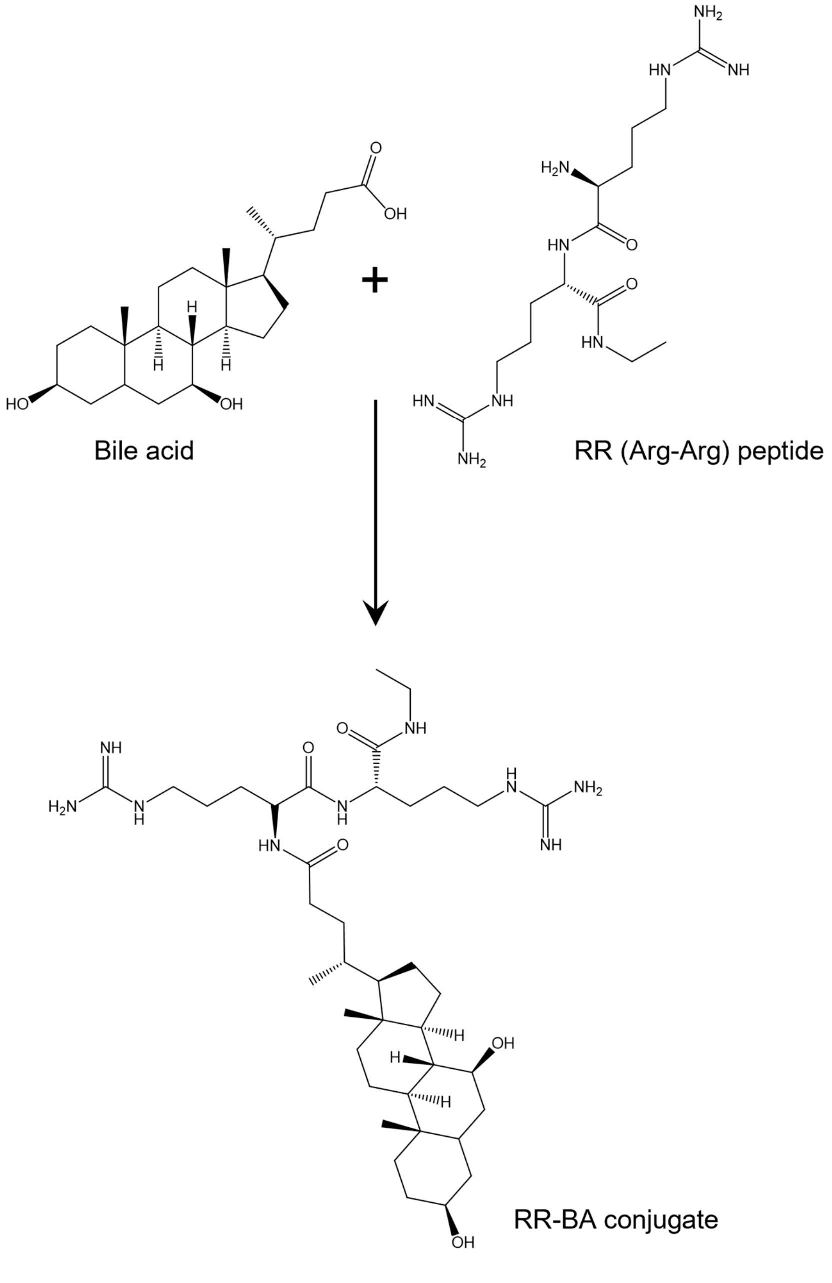

Figure S1. The chemical synthesis of RR-BA conjugate by using RR peptide and bile acid

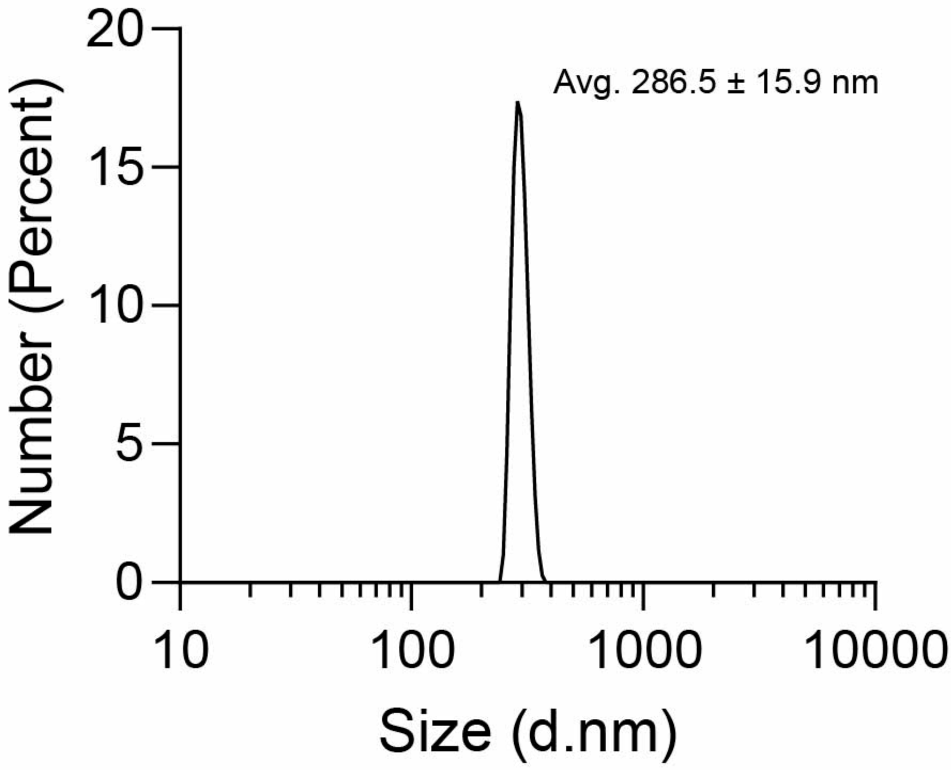

Figure S2. Size distribution analysis by dynamic light scattering (DLS). RR-BA were dispersed in normal saline (0.9% NaCl) thoroughly via sonication and vortexing, and RR-BA was measured at 1 mg/mL.

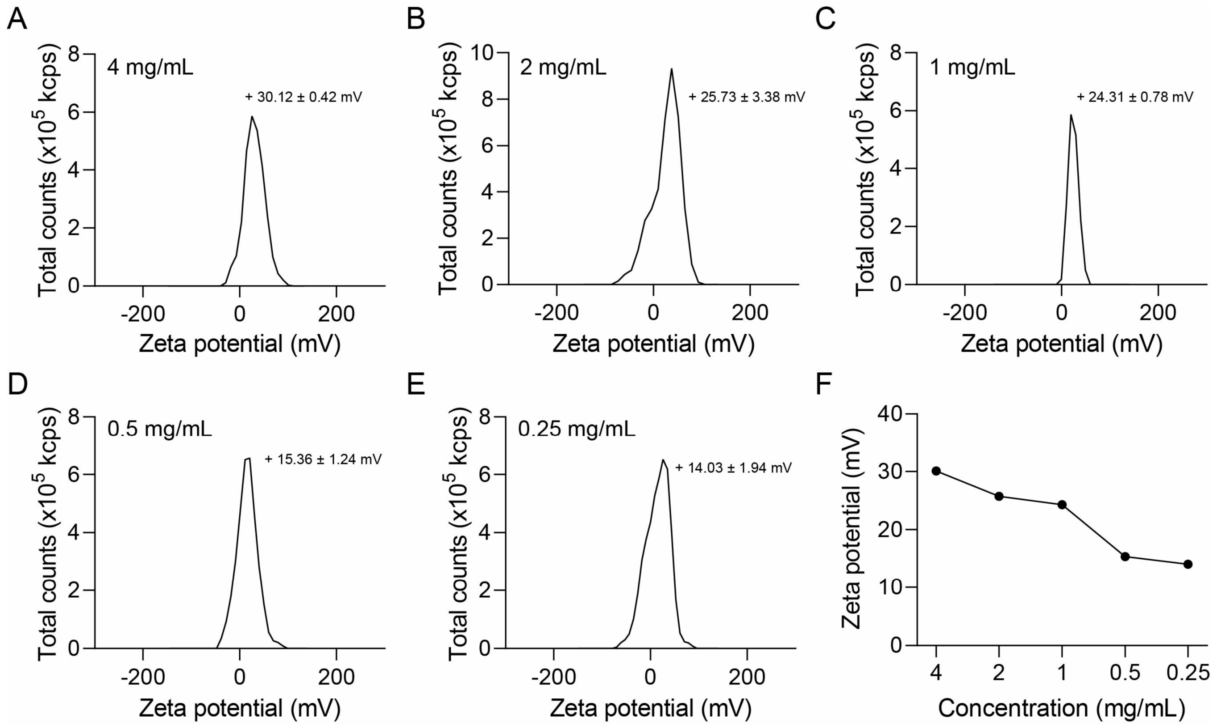

Figure S3. Determining the zeta potential of RR-BA. RR-BA is diluted in normal saline (0.9% NaCl) as in (A) 4 mg/mL, (B) 2 mg/mL, (C) 1 mg/mL, (D) 0.5 mg/mL and (E) 0.25 mg/mL final concentration. (F) Zeta potential value of various concentrations of RR-BA.

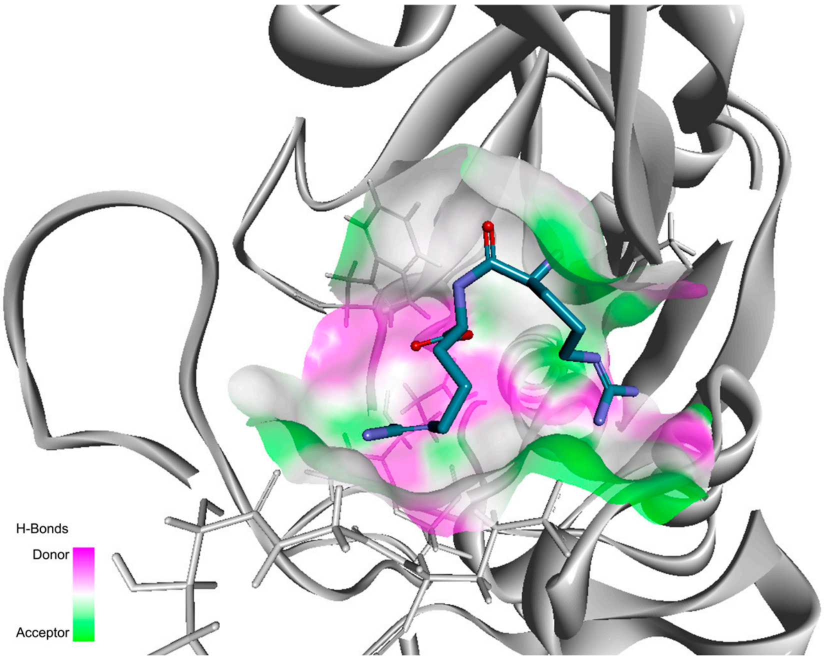

Figure S4. In silico docking position of RR-BA at cathepsin B binding site

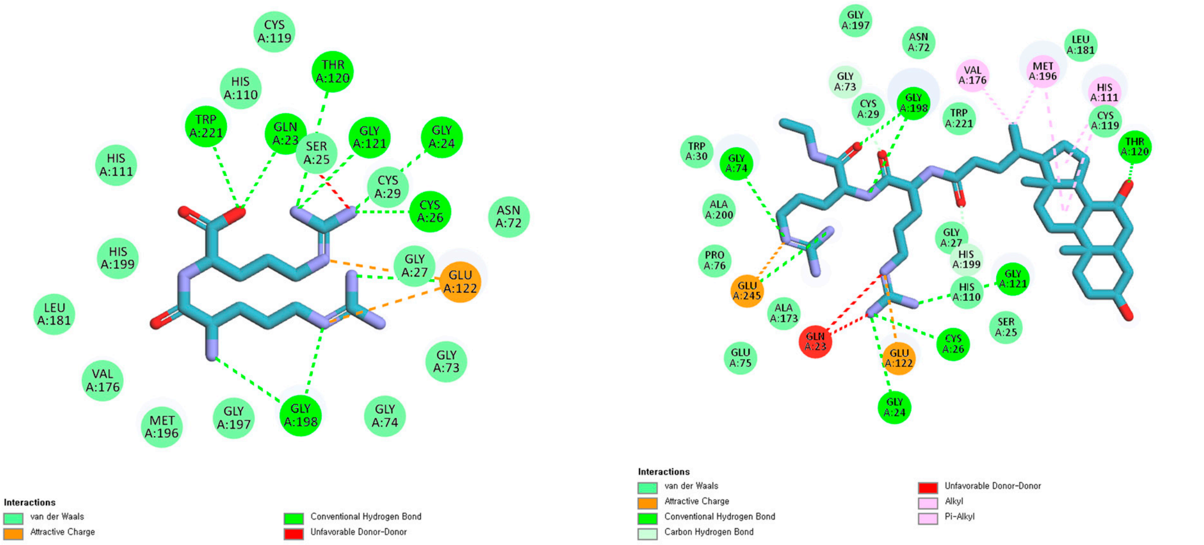

Figure S5. Molecular interaction analysis of RR peptide (left) and RR-BA (right) with cathepsin B
